# Spatially associated or composite life traces from Holocene paleosols and dune sands provide evidence for past biotic interactions

**DOI:** 10.1007/s00114-023-01837-w

**Published:** 2023-02-21

**Authors:** Shannon Hsieh, Alfred Uchman

**Affiliations:** Faculty of Geography and Geology, Institute of Geological Sciences, Jagiellonian University, Gronostajowa 3a, 30387 Kraków, Poland

**Keywords:** Ichnology, Neoichnology, Paleosol, Aeolian environment, Ecosystem engineering

## Abstract

Biotic interactions (e.g., predation, competition, commensalism) where organisms directly or indirectly influenced one another are of great interest to those studying the history of life but have been difficult to ascertain from fossils. Considering the usual caveats about the temporal resolution of paleontological data, traces and trace fossils in the sedimentary record can record co-occurrences of organisms or their behaviours with relatively high spatial fidelity in a location. Neoichnological studies and studies on recently buried traces, where direct trophic links or other connections between tracemakers are well-known, may help interpret when and where overlapping traces represented true biotic interactions. Examples from Holocene paleosols and other buried continental sediments in Poland include the tight association between mole and earthworm burrows, forming an ichnofabric representing a predator–prey relationship, and that of intersecting insect and root traces demonstrating the impact of trees as both ecosystem engineers and the basis for food chains. Trampling by ungulates, which leaves hoofprints and other sedimentary disturbances, may result in amensal or commensal effects on some biota in the short term and create heterogeneity that later trace-making organisms, such as invertebrate burrowers, can also respond to in turn, though such modified or composite traces may be challenging to interpret.

## Introduction

Organism-substrate interactions and organism-organism interactions that deliberately or incidentally modify substrates, resulting in structures in sediment such as trackways, nests, burrows, and root traces, interest both biological and geological researchers (Hansell 1993; Butler 1995; Buatois and Mángano 2011). Biologists may observe and study these in the context of their role in the organism’s life, as part of their extended phenotype, or as contributions to ecosystem engineering (Jones et al. 1994; Schaedelin and Taborsky 2009; Turner 2003, 2009). From a geological perspective, living organisms can be studied as geomorphic agents whose actions shape the physical landscape (Butler 1995; Stallins 2006; Bétard 2021). Preserved traces of life activity become ichnofossils (studied in the discipline of ichnology) which can be useful tools for reconstructing past environments farther back in time, especially in situations where body fossils may be absent or unsuited for providing information (Rhoads 1975; Crimes and Droser 1992; Minter et al. 2016; Buatois and Mángano 2018).

The degree to which biotic interactions (Boucot and Poinar 2010; Tevesz and McCall 2014; Fraser et al. 2021), such as predation (Kowalewski 2002; Kelley et al. 2003; Stanley 2008), competition (Sepkoski 1996; Taylor 2016; Liow et al. 2017; Klompmaker and Finnegan 2018), parasitism (Leung 2017; Baets et al. 2021), commensalism (Martinell and Domènech 2009; Zapalski 2011; Topper et al. 2014; Nanglu and Caron 2021) or amensalism (Thayer 1979; Buatois et al. 2018), and their impacts on ecological or evolutionary history can be inferred deep in the fossil record has long attracted scholarly debate. Direct comparisons of these interactions in the past and present are difficult due to the different types of evidence used from modern and fossil data, and the loss or modification of information through time. One challenge, among others, is that temporal or spatial resolutions are frequently not comparable to those used in modern ecological or biological research (Jablonski and Sepkoski 1996; Marenco and Hagadorn 2019). Physical mixing and time-averaging of fossil material may lead to erroneous assumptions that co-occurring individuals or species lived together or interacted directly when they did not. Though temporal resolution is still a constraint (only in rare situations can it be quite precise, such as Allport et al. (2022)’s finding of non-palimpsested *Skolithos* ichnofabrics in Carboniferous tidal rhymites confidently calibrated to up to 84 lunar days) and tracemaker identities are usually uncertain, trace fossils have an advantage over body fossils in that the fidelity of their relative spatial relationships is high. This is because structures such as footprints or burrows, though potentially distorted, are formed in situ and not transported as skeletons or shells may be (Crimes and Droser 1992).

Neoichnological approaches (Elders 1975; Bromley 1996; Dashtgard and Gingras 2012; Hembree 2016; Brewer and Falk 2021) utilize modern traces to help bridge the gap between observable biology in the present and ichnological data from the geological record. Many trace-making organisms from the Quaternary have biotic interactions which can either be observed today (if the species is still living) or easily inferred due to ample knowledge about their life habits or ecology; this provides a guide to understanding potentially analogous biotic interactions from the deeper past where such information is much sparser.

## Background

Neoichnological observations of inferred ongoing or recent past biotic interaction and ecosystem engineering were made in Holocene paleosols, inland dune sands, and other recently buried continental sedimentary deposits across Poland. Many of these are part of the European Sand Belt (ESB), a large depositional environment of late glacial and post-glacial aeolian sediments including dunes and sand sheets which were deposited starting around c. 15,500 cal year BP, later stabilized by forest vegetation and soil development, but then re-activated in some areas from human land use such as deforestation and grazing at various times throughout the Holocene (Tolksdorf and Kaiser 2012; Łapcik et al. 2021). Though mostly reforested by pine plantations in contemporary times, some small parts of the ESB are still incidentally or deliberately maintained as dune habitat or exposed drift sand.

The inland dune habitat, though nutrient-poor, along with other nearby areas of sandy soil, still supports across various successional stages a sizeable floral and faunal community ranging from grasses and trees to small invertebrates and large mammals that may either live there full-time, pass through from other, more resource-rich areas, or utilize the dunes for part of their life, for instance, digging in them for foraging and hibernation (Nijssen and Siepel 2010). A mosaic of environments represented by continental sediments which includes both well-developed forested areas with soil, anthropogenic habitats (e.g. agricultural fields and grazing land), and bare areas of fluvial or aeolian sediment such as those exposed on the surface in the ESB, contain a sizeable amount of biodiversity of possible tracemakers which interact with one another. In such environments, there is a potential to record a great variety of traces (representing different organisms and their behaviors) that may also overlap or interact on a diversity of spatial scales, due to the reasonably high surface area and volume of sediment receptive to registering and preserving them. These traces may be quite abundant and easily recognized on present-day surfaces in addition to buried sand or paleosol that reflects their longer-term Holocene history.

## Soil-earthworm-mole food chain reflected through spatially co-occurring burrows

**Fig. 1:**
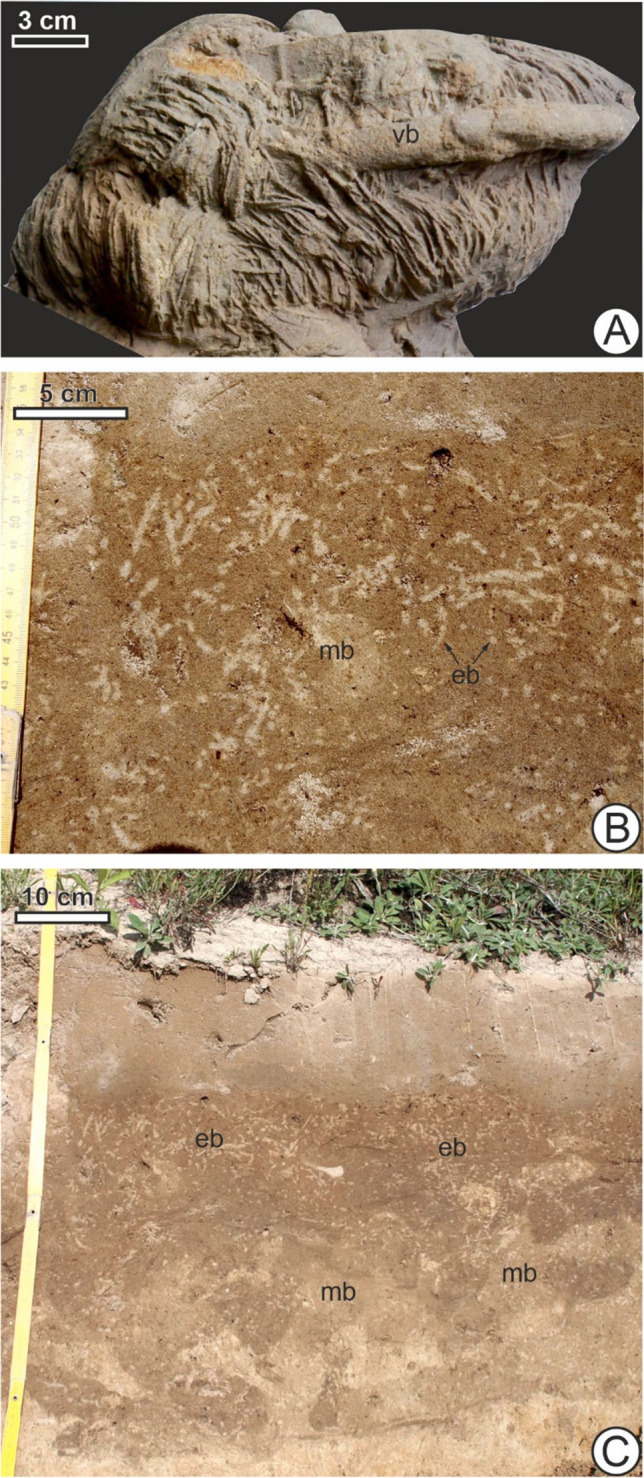
Potential evidence of analogous predator–prey interactions in fossil and subfossil form. **A** An example of the marine trace fossil *Rusophycus dispar* overlapping with a vermiform burrow (vb) from the Mickwitzia Sandstone (lower Cambrian), Kinnekule, Sweden (speciemen X713, Swedish Museum of Natural History, Stockholm), which has been argued as evidence that their trace-makers, trilobites, interacted with and preyed on worms, early in the history of animal life. **B**, **C** Excavated horizons near Rakszawa Zakład Górniczy (mining plant), in south-eastern Poland, reveal an ichnofabric of European mole (mb) and earthworm burrows (eb) in close proximity in an area of sandy paleosol where there is sufficient organic matter (A—horizontal section; B—vertical section)

Reconstruction of ancient food chains and food webs for fossil ecosystems has been a goal of palaeontologists, with detailed examples coming from periods as early as the Cambrian (Butterfield 2001; Dunne et al. 2008; Shaw et al. 2021) using various lines of evidence. Though on rare occasions body fossils can show “frozen behavior” (Boucot and Poinar 2010) such as fish caught in the act of swallowing other fish (Grande 2013), trace fossils are a major source of evidence for many trophic interactions, with predatory traces of biting or drilling on skeletons or shells being especially well-documented (Kelley et al. 2003). Predatory encounters based on intersecting traces in sediment are extremely rare and desired subjects of study but can be disputed and difficult to interpret – among the few proposed cases in the academic literature are trackways of tetrapods chasing and catching other tetrapods or invertebrates (Lockley and Madsen 1993; Hunt and Lucas 1998) and trilobites, as inferred from trace fossils such as *Rusophycus* or *Cruziana*, preying on infaunal marine worms (Jensen 1990; Brandt et al. 1995; Rydell et al. 2001; Carvalho 2006; Selly et al. 2016; Fig. 1a). Situations where both predators and prey are comparatively fast and motile, engaging in surface locomotion and able to range widely across an area, as with footprints of tetrapods chasing other tetrapods, might make captured brief interactions extremely rare (Lockley and Madsen 1993; Hunt and Lucas 1998) in contrast to ones in which the interactions are with predators seeking out much slower, sedentary or spatially concentrated prey with limited mobility which, on one extreme, more closely resembles foraging on relatively static or immotile resources such as deposit feeding or grazing. The latter would have a more easily recognizable ichnofossil record. Evidence of predation on burrowing animals may in some cases be similar to the latter, being variable in terms of preservational potential. These may include Selly et al. (2016)’s findings that *Rusophycus* intersected vermiform burrows more often than expected by chance and that positive size correlations between paired traces suggest size-selective predation by the trilobite tracemaker. Falk et al. (2010) described feeding traces interpreted as probe marks and peck marks, associated with bird tracks from the Cretaceous, which were also found in association with invertebrate traces including *Cochlichnus*, *Steinichnus*, and *Arenicolites*. They suggested that the invertebrate traces, found to both cross-cut the bird tracks as well as being cross-cut by them, represented those of potential prey that the avian tracemakers were searching and hunting for. Some fossil bird tracks and traces (e.g., Lockley et al. 2009; Lockley and Harris 2010; Falk et al. 2014) were examined and noted as being remarkably similar to those produced by modern birds hunting burrowing invertebrates, with examples that may represent shuffling gaits possibly linked to “foot-stirring” foraging behavior (Lockley et al. 2009) as well as those resembling scything feeding traces from extant spoonbills (Falk et al. 2014).

Earthworms consume decaying organic material (Curry and Schmidt 2007) and have long been an important part of detritus-based food chains in terrestrial ecosystems. Knowledge about their deep-time history is hindered by their extremely poor body fossil record as soft-bodied animals, though various ichnofossils have been attributed to them (Hazen 1937; Verde et al. 2007; Chin et al. 2013; Shcherbakov et al. 2020). Chin et al. (2013), for instance, found closely packed, criss-crossing networks of burrows resembling those of modern earthworms just above the Cretaceous-Paleogene boundary. They considered that these may reflect their tracemakers thriving in soil despite the drop in primary productivity associated with the end-Cretaceous extinction, and that abundant populations of earthworms could have played a critical role in allowing the survival and success of carnivorous land vertebrates such as mammals and birds through detritus-based food chains into the early Paleocene.

Although hundreds of species of terrestrial vertebrates today including amphibians, reptiles, birds, and mammals will eat earthworms (Macdonald 1983), their relation to one specialized predator, the mole, leads to a particularly strong signal in the soil or sediment, due to both predator and prey being abundant burrowers with subterranean lifestyles as well as an extreme dietary dependency requiring proximity of one to the other. The European mole, *Talpa europaea* Linnaeus 1758, has been reported to feed predominantly or nearly exclusively on earthworms depending on their availability so that they make up half or more, sometimes close to 100%, of its diet by weight (Mellanby 1966; Raw 1966; Funmilayo 1979; Edwards 2004). Skoczeń (1966) found that 82.7–100% of mole stomachs contained earthworms in a sample from the grasslands of the Kraków area and mountainous regions of southern Poland. This dependency is also evident in patterns of distribution and population densities of the two animals. For instance, Funmilayo (1977)’s research in Southeast Scotland found that in addition to living in the areas with the highest earthworm density and biomass, moles also distributed themselves vertically according to where this prey source could be found, tunneling deeply where deep-burrowing species were present but making shallow runs where earthworms were near the surface. Funmilayo (1977) found that physical factors such as soil type and altitude did not significantly influence the moles’ distribution directly but because these factors limited their prey’s abundance, their own abundance was indirectly affected.

A mole tunnel intersecting areas of earthworm burrows as observed in Fig. 1B, C may potentially record moments of predation, but even if such an individual event cannot be easily recognized, repeated spatial co-occurrence and overlap of the two tracemakers’ burrows more broadly is evidence of a strong trophic link which involves physical proximity and very frequent encounter and predation, since moles may eat as much as their own body weight in a day (e.g. research by Catania (2008) has shown that an eastern American mole *Scalopus aquaticus* Linnaeus 1758 could eat an average of 23 worms a day in captivity). Earthworms have an escape response to foraging moles, fleeing to the surface in response to vibrations (Catania 2008). Moles have been known to make caches of live earthworms by biting their anterior segments and incapacitating them so that they do not burrow away and can be kept as surplus (Raw 1966); as many as 470 worms in a cache, with a total weight of 820 g, was found by Skoczeń (1970). Earlier, Barrett-Hamilton (1910) had wondered if putative caches were worms having fallen into mole tunnels before later research confirmed the nature of this type of storage (as reviewed in Macdonald 1983), indicating that these represent a direct interaction between the animals. The discovery of this caching behavior is an example of how insights about interactions between living tracemakers can follow from initial observations and hypotheses about spatial associations between them and/or their traces in soils or sediment.

Though ichnofossil records of direct predation in burrows or trackways are likely to be extremely rare and difficult to infer in general, often reliant on interpretations of the way trails or burrows terminate and intersect (Hunt and Lucas 1998; Lockley and Madsen 1993; Jensen 1990; Brandt et al. 1995; Rydell et al. 2001; Carvalho 2006; Selly et al. 2016), Holocene subfossil earthworm-mole tunnel ichnofabrics (Fig. 1B, C) demonstrate that spatial proximity and repeated co-occurrence can in some situations provide support for past predator–prey relationships especially if there is prior knowledge or reasonable assumption based on the tracemakers’ functional morphology, gut contents, or other biological data. The availability of this background knowledge varies widely between tracemakers and trace-making taxa. In the case of talpids (the mole family), a body fossil record is known as far back as the Eocene, which also sheds light on the evolution of fossoriality in the group (Voorhies 1977; Gobetz 2005; Lloyd and Eberle 2008; Schwermann and Thompson 2015). Gobetz (2005) studied modern foraging tunnels from eastern American moles based on casts, finding features such as claw impressions which might allow attribution of potentially similar-looking fossil burrows to extinct moles or mole-like burrowing locomotion. Spatial co-occurrence of these traces with those of potential earthworm or other burrowing invertebrate prey may potentially exist in the Cenozoic ichnofossil record.

## Trees as keystone species and the ichnological record of root-invertebrate interactions

Trees have been described as keystone species (Bond 1994), having disproportionate influence on their surroundings and on other species in many ecosystems (Munzbergova and Ward 2002; Kivinen et al. 2020). Individual isolated trees may be especially important for providing microhabitats and micro-climates and supporting local ecological functions in places where they are scattered across the landscape with open areas in between (Manning et al. 2006). This would be true in the ESB where Scots pine *Pinus sylvestris* L. occurs in early successional stages on dunes; these pines are often deliberately planted as part of monocultures (Sewerniak and Jankowski 2017) but natural colonization also happens there since the region is part of its native range (Krakau et al. 2013).

**Fig. 2:**
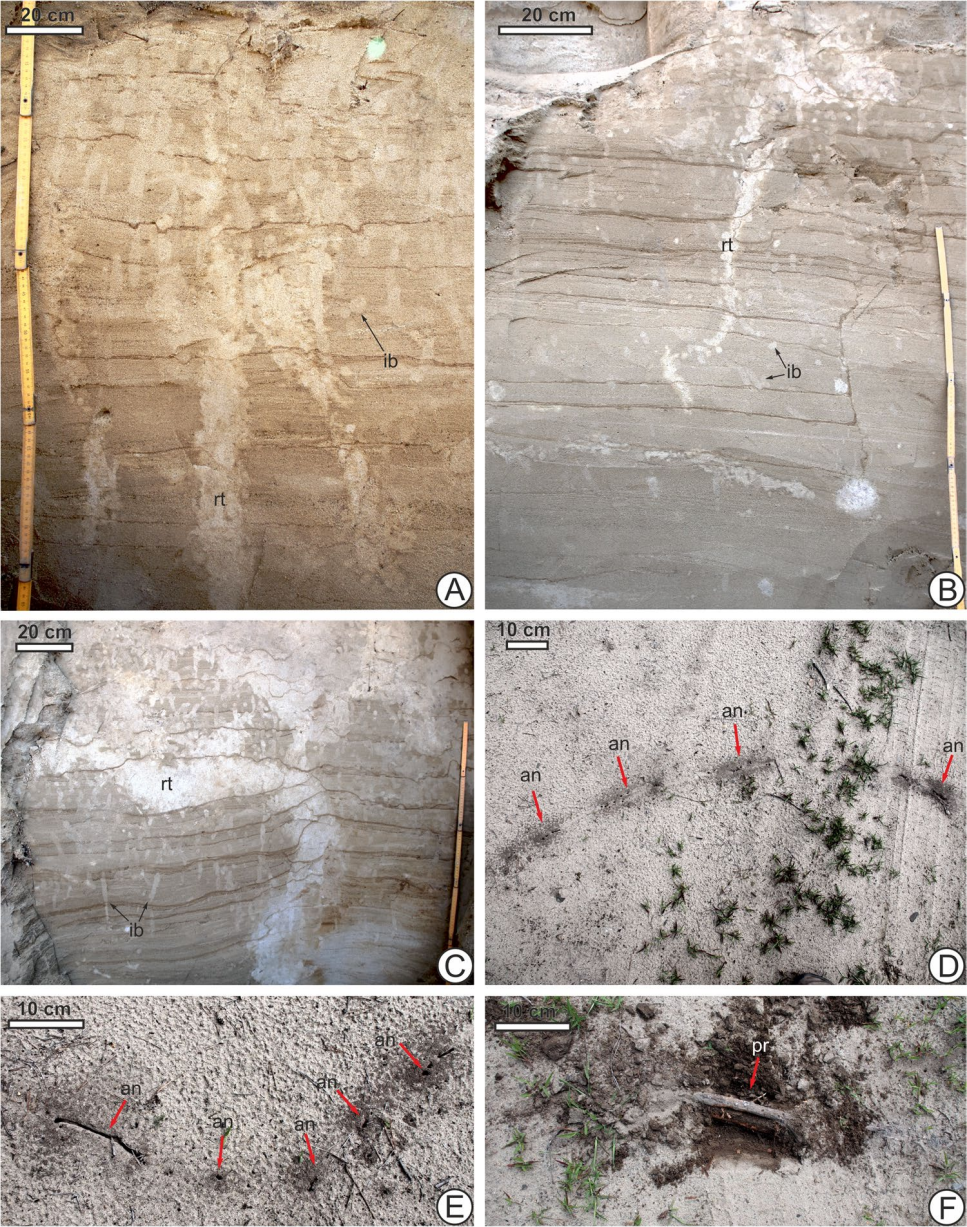
Evidence of Scots pine *Pinus sylvestris*, L., as a keystone species, based on life traces in dune sediment. Vertical section of dune sand layers showing subfossil pine root traces (rt) or casts with high densities of insect burrows (ib), both intersecting and surrounding them, at Smolarzyny (**A**) and Żarówka (**B**, **C**) in south-eastern Poland; this can represent animals dependent on the tree for resources. **D, E** Ant nests (an) at Smolarzyny made along a living pine root (course indicated by arrows) on the surface of a dune show a tight spatial coupling, in horizontal view, between two identifiable tracemakers, one animal and one plant. **F** Excavated shallow pine root (pr, arrow) associated with ant nests presented in **D** and **E**

Roots of Scots pine can descend to 4 m below the surface (Uchman et al., in prep) and provide food and shelter to many animals, especially insects. For instance, grubs of the scarab beetle *Polyphylla fullo* L., and May beetle *Melolontha melolontha* L. consume pine roots while the omnivorous ground beetle *Harpalus rufipex* (Degeer) consumes seeds, including those of conifers. These insects may have left the abundant traces seen in Fig. 2A, B, C. Pine roots can be solid objects with surfaces where it may be physically easy to build burrows and nests—for instance, ant nests are spatially arranged along a root as shown in Fig. 2D, E, F. The ants may also nest around the roots due to the food resources available, including prey insects attracted by them, demonstrating both the trophic and non-trophic importance of trees in the ecosystem. Additionally, after the roots are dead or decaying other insects may feed on the organic matter and utilize the empty spaces or loose sediments where they once were. How many species or how large of a population of animals is directly or indirectly dependant on a particular tree’s roots (or those of a single species of tree) is often difficult to determine with data from living systems, let alone subfossil or fossil ones. Nonetheless, neoichnological observations provide clues about the trees’ outsized importance.

High densities of invertebrate burrows close to or overlapping with root traces or casts of roots, with diminishing burrow densities or lack of burrows farther away, may be neoichnological evidence of the trees’ role in the community, historically, even if the tracemakers leave no bodily remains (Fig. 2b). Although the full shapes of burrows are not easily observed in loose unconsolidated sediments like dune sands with individual horizontal or vertical sections alone, some variability such as U-shapes alongside finger-like or linear burrows may be observed. It is possible that ichnofabric indices (Droser and Bottjer 1993), ichnodisparity, and ichnodiversity (Buatois and Mángano 2013), measures traditionally used for fossil assemblages (usually in the marine realm), may be used to infer the presence of keystone species and biotic interactions with them in the terrestrial continental realm. The existence of a keystone species such as the Scots pine on dune sands, whose presence allows the survival of many other organisms that leave their own traces, may be inferred in the form of high ichnofabric indices in proximity to root casts, as well as possible high ichnodiversity or ichnodisparity (in situations where trace-making insect diversity is high or behavioral variability is high as the insects feed, shelter, pupate, brood offspring and perform other actions on or in proximity to the roots) being recorded in areas where the pine’s root traces are present. Low values of these measures would then be expected to be found where the pines are absent or very far away.

**Fig. 3:**
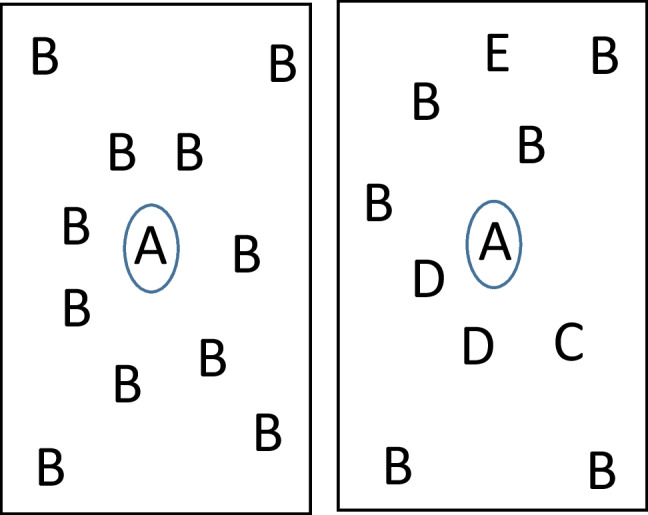
Schematic diagram of theoretical spatial associations if the tracemaker producing a trace, A, is important as a keystone species providing food and shelter for other tracemakers, with letters representing occurrences of ichnospecies or ichnotaxa in vertical or horizontal view. (Left) There may be a high abundance of other traces near A. (Right) There may be a high diversity of other traces near A

Indeed, an intriguing but speculative possibility for ichnological and neoichnological research is to examine hypotheses about or attempt to infer possibly previously unrecognized examples of keystone taxa, keystone functional groups, or ecosystem engineers by patterns of co-occurence from traces, which in the modern can then be compared or tested with biological observations where living examples still exist. For instance, if traces generally spatially cluster around A, with diminishing trace abundance or diversity farther away from A, then A could be a candidate for a keystone taxon (Fig. 3), among other explanations. Although it may be difficult to prove the presence of one tracemaker is truly required for or facilitates the survival or persistence of other, often, later tracemakers (as opposed to them simply being earlier to colonize and being the first in an area without having later species rely on them), modern examples such as with pine root-feeding insects can provide identifiable situations where these inferences of dependency or disproportionate impact may be justified.

## Interactions between large vertebrate trampling and ground-dwelling or burrowing biota

**Fig. 4:**
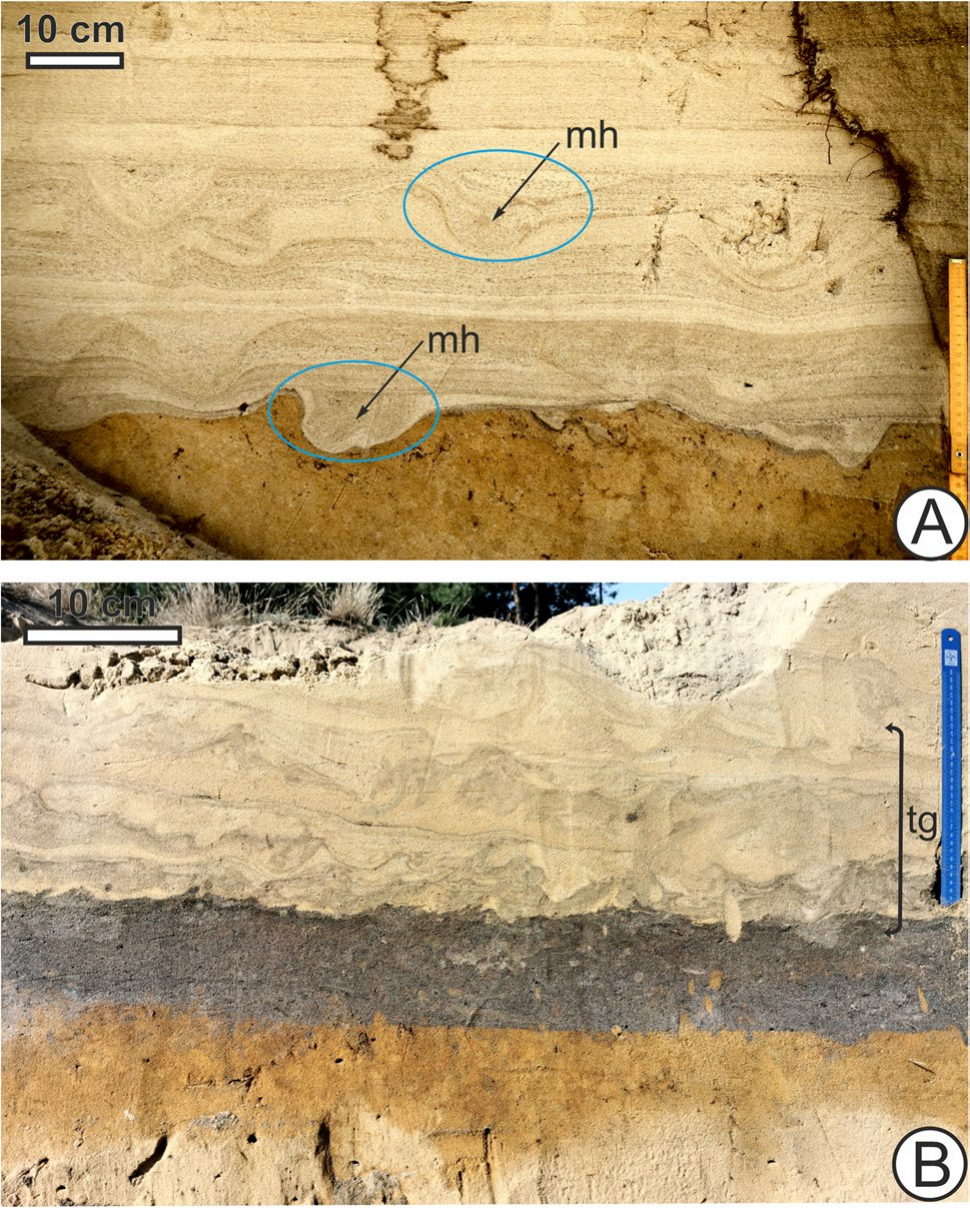
Since intense trampling on dunes by large mammals disturbs or negatively impacts small burrowers (amensalism), one would predict for instance that small mammal or invertebrate burrows (Fig. 1) are less commonly found in horizons that show evidence of high densities of mammal hoofprints (mh) or footprints as seen in **A** (arrows), near Unin, Masovian Voivodeship, east-central Poland, or trample grounds (tg) as seen in **B** (layer of heavily trampled sand above a paleosol (bracket), likely resulting from cattle grazing, at Oddziały, south-central Poland)

Until recent historic times, the European bison *Bison bonasus* L., aurochs *Bos primigenius* Bojanus, and tarpan horse *Equus ferus ferus* Boddaert were large grazers (Cromsigt et al. 2018) living in the ESB with the latter two, wild relatives of domestic cattle and horse, becoming extinct in the seventeenth and the twentieth century, respectively, due to human activity. Grazing by large mammals, both wild and domestic, has kept coastal and inland dune habitats open and sparsely vegetated (Tolksdorf and Kaiser 2012; Brunbjerg et al. 2014; Valdés-Correcher et al. 2018; Prach et al. 2021) where conditions would otherwise favour closed canopy forest development. Thus, large mammals may be zoogeomorphic agents responsible for maintaining the dune habitat itself (Butler 1995). Trampling has left evidence of disturbed horizons with high densities of probable domestic cattle hoofprints and those of other ungulates within layers of dune sand across centuries and millennia (Hsieh et al. 2023). Due to restricted movement from fencing or tethering, tracks of livestock kept by humans may be denser and associated with bioturbation more intensive than those of wild animals, and this effect would have already influenced many places around the world in the late Holocene. In addition to the vegetation-removal effects of grazing, burrowing or ground-dwelling invertebrates and other biota may be negatively impacted by the physical act of trampling, as a form of amensalism (Duncan and Holdaway 1989; Bates et al. 2007; Schlacher et al. 2016; Andriuzzi and Wall 2018); for instance, Beever and Herrick (2006) found, in the western US, higher ant mound abundance at sites where horses had been removed compared to horse-occupied sites and suggested that direct trampling of mounds and soil compaction, among other factors, may be an influence. Soil fauna such as earthworms and moles may have more difficulty surviving in areas with grazing cattle, with those near the surface most affected (Trimble and Mendel 1995). Amensalism by animals disturbing sedimentary layers and surfaces from either above or below would be expected to occur throughout the biosphere across terrestrial and marine communities alike and has been suggested to be ecologically important as far back as the Ediacaran-Cambrian, over half a billion years ago, when early bioturbators negatively impacted biota living previously unperturbed on the seafloor (Buatois et al. 2018). Specifically, in continental settings, one might expect the ichnological record of amensalism resulting from trampling to appear as negative co-occurrence patterns between large mammal footprints or tracks and small invertebrate traces or burrows on surfaces and within individual sedimentary layers—one might be expected to be found abundantly where the other is sparse (Fig. 4). The effect of direct physical disturbance may be difficult to disentangle from the effect caused by destruction of resources (e.g. vegetation removal or topsoil erosion) from the animals trampling and grazing, however.

On the other hand, when these disturbances have passed or are no longer significant, small burrowers may interact with, penetrate, and cross-cut previously heavily trampled layers or those containing sedimentary structures such as hoofprints. The effects produced by the presence and locomotion of large mammals on open ground can have a positive impact (commensalism) on smaller fauna. For instance, grazing and trampling cattle remove vegetation and maintain open habitats which benefit and sustain tiger beetle populations, even considering losses that result from cattle hooves cutting into their larval burrows (Knisley 2011). Wallowing or trampling by ungulates such as bovids and wild boar can create pools of water that are needed by amphibians or aquatic insects (Williams 1997; Baruzzi and Krofel 2017). Cattle hoof prints on slopes can also catch seeds and prevent their transport downhill by water runoff, thus influencing plant establishment (Isselin-Nondedeu et al. 2006). In Casa Grande, Arizona, on land used for grazing, new queens of the ant species *Veromessor pergandei* Mayr will gather in cattle hoof prints after their mating flight, preferring to dig their first nests in the shade provided by these depressions in sand (Kwapich, C., personal comm.). Therefore, large ungulate traces may be linked to disturbance and negative effects on smaller biota in the short term but on longer time scales, when the disturbance is gone, lead to biodiversity-promoting heterogeneity. Nickell et al. (2018) found that while active wallowing by North American bison reduced the biodiversity and abundance of arthropods, later abandoned wallows could increase local arthropod diversity and seasonal abundance by providing a distinct microhabitat on the prairie. European bison may potentially have similar effects. Traditionally considered a forest species based on refugial populations (e.g., in the Białowieża Primeval Forest) resulting from human activity, based on isotope data, it was found to previously occupy more open habitats in the early Holocene (Bocherens et al. 2015) and presently, coastal dunes are among the habitats it has been reintroduced into (Valdés-Correcher et al. 2018; Pedersen et al. 2019). North American bison have a well-documented history of occupying and disturbing inland dunefield habitats through the Holocene as summarized by Fox et al. (2012), including deliberate wallows in sand that are used successively, modifying the landscape (Soper 1941; Potyondi 1995; Geist 1996; Bowyer et al. 1998; Coppedge et al. 1999), raising the possibility that European bison could have acted similarly in the ESB and that these structures produced by them may have been or still be present. The physical traces and topographical influence of wallowing by large fauna such as bison or wild boar may persist long past the life of the animal but may be difficult to distinguish from erosional structures produced by abiotic forces, long-term in the geological record. Indeed, one problem previously noted with regard to the sedimentary and stratigraphic record is the underdetermination of biological influence—geomorphic processes or sedimentary structures triggered or produced by living organisms may not be easily distinguished by those caused by solely physical processes (Davies et al. 2020).

**Fig. 5:**
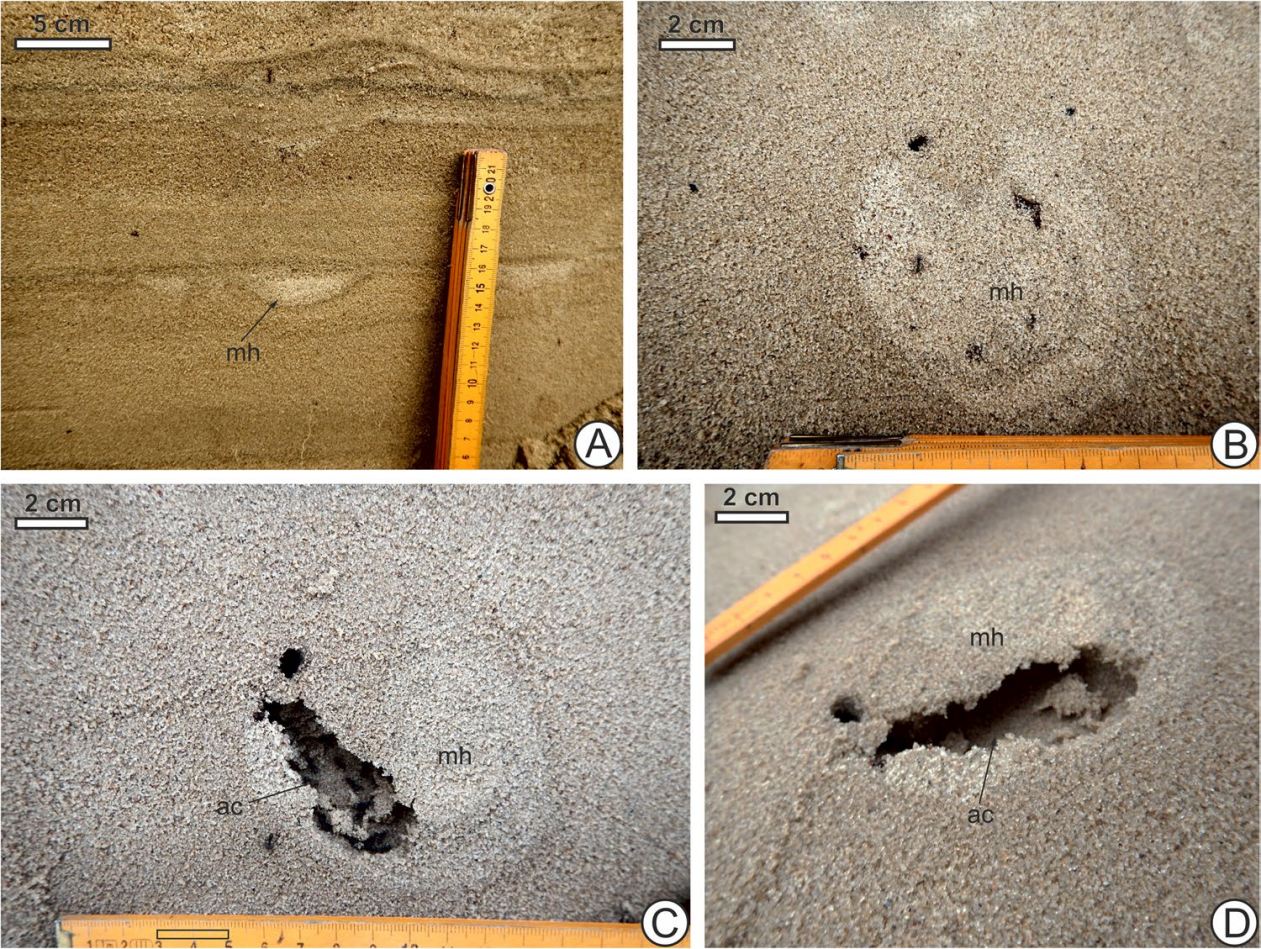
Images of excavated buried footprints, including one example that contained nesting ants inside near Ochotnik, central Poland. **A** Vertical cross section of horizons of dune sand containing mammal hoofprints (mh) or footprints, visible as deformations in the layers filled with sands of contrasting color or lithology. **B** Excavated horizontal cross section of a particular buried hoofprint which contained a chamber made by ants inside it. **C**, **D** Chamber (ac) inside horizontally sectioned footprint in **B** with ants photographed inside

Burrowing insects may interact with heterogeneity resulting from traces such as footprints which have been infilled and are long-buried rather than present at the surface. Near the village of Ochotnik in central Poland, excavation and horizontal sectioning of layers containing ungulate tracks (Fig. 5a) inadvertently revealed a chamber containing multiple ants inside an individual hoof print (Fig. 5b–d) that was infilled with sand of somewhat different colour and lithology than the surrounding layers. The hoof print, likely from cattle, was in a horizon whose closest underlying paleosol was radiocarbon-dated to possibly as old as the fifteenth century AD (Hsieh et al. 2023) and thus would have been remained possibly buried for centuries before being penetrated by the living ants and having the sediment within it reworked. The boundaries of the ants’ chamber appear to be potentially influenced by the edges or outline of the footprint (Fig. 5c, d). Minter et al. (2012) showed that environmental features such as planes between sediment layers influence ants to tunnel horizontally along them, so a similar phenomenon may have happened here if the infill was preferentially excavated compared to walls of compacted sediment along the hoof print. Since tracks act as depressions that collect material contrasting lithographically with surrounding layers (Buynevich 2011a, 2011b, 2012, 2015, 2020), burrowers such as ants may also respond to these properties of the infill as they are known to be sensitive to sediment properties such as grain size, coarseness, or cohesiveness when engaging in locomotion and nest-building (Aleksiev et al. 2007; Bernadou and Fourcassié 2008; Toffin et al. 2010).

In the above example, there is no evidence that the influence of the past trackmaker on the ants, even if it did influence their burrowing patterns, was strongly positive or negative. Many examples have been studied where animal-produced structures have long-lasting impacts in terms of survival or fitness for later individuals who never interacted with their makers (e.g., Fisher at el. 2019); and old nests, cavities, and burrows, such as badger setts, for instance, may be remodelled and re-used for centuries (Roper 1992), outlasting their initial producers. However, it remains an open question if these effects would be inferable from fossilized traces deeper in geological time as with, for example, inferred composite or compound trace fossils.

## Discussion and conclusions

### Biologically driven heterogeneity and self-organizational feedbacks

**Fig. 6:**
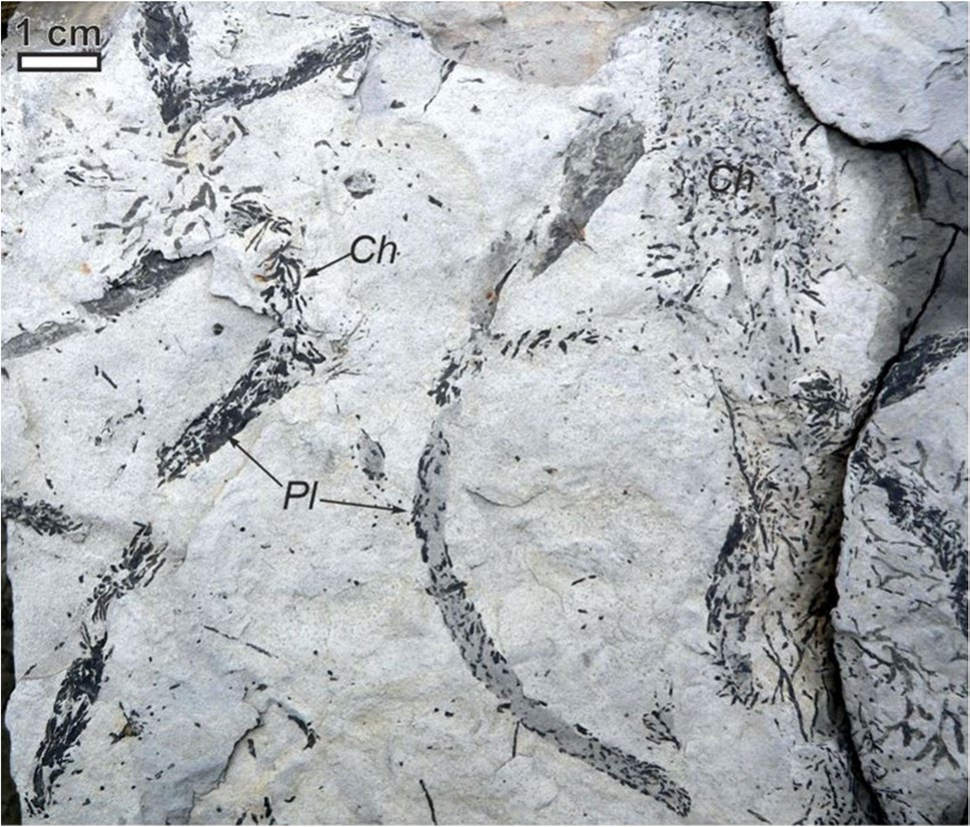
Fossil burrows, *Chondrites* (*Ch*) preferentially produced within the filling of *Planolites* (*Pl*), from the Siliceous Marl (Turonian) at Rzyki, Silesian Nape, Carpathians, southern Poland. These trace fossils may represent their tracemakers, possibly worm-like marine animals, seeking out concentrations of material or microhabitats created by the effects of the earlier tracemakers

Three previously mentioned examples from Holocene dunes in the ESB—mole and earthworm tunnels, root traces and insect burrows, and ant chambers within buried ungulate tracks involve some form of trace-making activity spatially co-occurring with or modifying one another. Self-organizing feedbacks where organisms concentrate their trace-making in places others have previously made their own traces are easily seen today and would have existed early in the history of life. For instance, humans and animals on land frequently follow and repeatedly reinforce paths such as game trails and dirt roads already created by others, as they can be more compact or free of obstacles and thus more efficient to travel on (e.g. Whiteman and Buskirk 2013; Whitridge 2014; Kolowski and Forrester 2017). The roots of plants often follow previous generations of root growth which had penetrated the substrate earlier or taken advantage of gaps created by abiotic processes like ice wedging initially (Uchman et al., in prep.). Similarly, during the Ediacaran-Cambrian transition, seafloor burrowing by early animals promoted irrigation, organic matter mixing, and other effects that encouraged even more burrowing (McIlroy and Logan 1999). Burrowing has been considered among the most energetically costly forms of animal locomotion, much more than walking, swimming, or flying (Wu et al. 2015) so it is reasonable to expect animals, in addition to plants, to take paths of least resistance such as where sediment has been loosened by others. There are other additional benefits, such as pre-existing burrows being able to fill with material (e.g. organic or food-rich sediment) desired by the later tracemakers. An example observed from the fossil record where earlier tracemakers may influence the arrival and distribution of later tracemakers is seen in the trace fossil *Chondrites* with burrows preferentially concentrated in the filling of *Planolites* (Fig. 6). Re-using or occupying and adding to old or abandoned structures so that they are active and persist a very long time, even if their morphology changes, is common. In modern continental settings, burrow complexity often increases with time of occupation in addition to increasing sociality or higher numbers of co-inhabitants in the same place, with evidence of this commonly observed from tetrapods and terrestrial arthropods (Roper 1992; Hansell 1993; Ziege et al. 2015; Hembree 2016).

Trace density or distribution often reflects resource availability (Koy and Plotnick 2010), so increasing density or co-occurrence of traces may also reflect biotic interactions in situations where animals either exploit the same shared resources (in some cases demonstrating possible competition, such as the crowded burrows around the pine root in Fig. 2A–C) or exploit or rely on one another for resources (e.g. predation, commensalism) as possibly with the ant nests near the root in Fig. 2D–F. This is exemplified in the strong association between the Scots pine and insect burrows seen throughout dunes in Poland and other parts of the ESB. The savannah hypothesis of Budd and Jensen (2017) for the Ediacaran-Cambrian transition, drawing an analogy to the role of the heterogeneous savannah in human evolution, proposed that the breaking of uniformity of organic carbon availability into areas of concentrated resources spurred early bilaterian animal evolution. History would seem to repeat itself regularly on much smaller temporal or spatial scales in situations regularly studied by ecologists as primary or secondary succession (Chang and Turner 2019), where flora and fauna colonize barren, and relatively homogenous, substrates such as dunes so that their surfaces and subsurface layers could be rendered increasingly more heterogeneous from biological activity. Early successional habitats where identifiable trace-makers arrive and begin ecosystem engineering processes that allow recruitment of other tracemakers would thus be good places for neoichnological observations on biologically driven heterogeneity and self-organizational feedbacks.

### Lessons for interpretation of biotic interactions from fossils

These modern or Holocene examples of co-occurring traces in inland dunes demonstrate evidence of biotic interactions or trophic relationships, based on knowledge of their living tracemakers. How well these interactions can be inferred from spatial proximity has long been debated by palaeontologists and ecologists alike. Zapalski (2011), for instance, argued that commensalism is barely detectable from modern, let alone ancient, associations due to the improbability of strictly neutral outcomes versus mildly positive or negative ones, and that the information needed to prove them is difficult to gather and especially unavailable from fossils (e.g. biochemical changes which leave little impact on broader, external morphology). Analyses of modern co-occurring or spatially associating taxa in general are popular in systems ranging from ocean ecosystems (e.g. Ainley et al. 2009) to soils (Goberna and Verdú 2022), but have been critiqued as producing inaccurate interpretations (Blanchet et al. 2020; Goberna and Verdú 2022) for a number of reasons. Ability to detect interactions can be scale-dependent—for example, when viewed on finer spatial scales, prey may be negatively associated with predators as they avoid them, but a positive spatial association may be seen when looking at larger spatial scales, from the latter tracking or following the former (Thurman et al. 2019). Ichnological analyses using ancient bedding planes also need to consider heterogeneity at different scales, where traces may appear clustered together when viewed at one resolution but not another; for instance, Marenco and Hagadorn (2019) noted that many studies look at bedding planes of limited horizontal extent, typically 1 m^2^ or smaller, with exposures of 2 m^2^ being uncommon, and consider that larger surfaces or combined samples may be needed to make broad paleoecological interpretations.

Another common issue is inability to distinguish between associations of organisms that truly interact as opposed to respond to commonalities in environmental or settlement preferences, whenever either their bodies, living or dead, or their traces co-occur. Freilich et al. (2018), looking at modern data from the rocky intertidal of Chile, find that co-occurrence provided more information about the effects of environmental conditions than biotic interactions, and that among the latter niche-expanding non-trophic interactions tended to be ones that leave more recognizable signals because they create or expand niche spaces for those they interact with. These authors consider that this might not reflect biotic interactions being less ecologically important, but rather more difficult to detect with these types of data. For comparison, it is notable that trace fossil research has traditionally had a long-standing focus, as with the ichnofacies paradigm (MacEachern et al. 2007), on environmental controls such as temperature, physical conditions, and organic resource availability, which tends to be more reliably inferred from records in deep time, over interpretation of biotic interaction, such as the presence of predators or competitors. Nonetheless, as many traces from the Holocene soils, including these on continental inland dunes, show, both biotic interactions (e.g. the European mole’s trophic dependency on earthworms influencing its burrow locations) and physical or environmental conditions (e.g. both animals’ burrowing habits being influenced by the below-ground freezing line and earthworms’ burrows being controlled by organic matter availability) can play a big role in ichnofabric composition, and biotic interactions may be interpretable in situations where tracemaker life habits are well-studied and understood.

Kidwell and Tomasovych (2013), discussed how time-averaging in death assemblages, accumulated remains which are precursors to buried fossil assemblages, leads to a loss of the finer resolution that can be seen with living assemblages but also allows insights into long-term metacommunity structure and other information that may be missed with studies on shorter timescales typically examined by ecologists. Similar advantages may also come from examining recently buried or subfossil trace, rather than skeletal, assemblages. For instance, although ichnofabrics in dune sands or paleosols may not allow recognition of any one individual predation event by a mole on an earthworm or indicate that any one single burrowing insect was dependant on a particular tree root, seeing co-occurring traces persistent or widespread in time and place may hint at, if not validate, possible true long-lasting biotic associations that both happened in the recent past and are important in a community today. Examples of past biotic interactions more heavily studied with ichnofossils have been those taking place on or within leaves (such as with leaf-mining insects; Labandeira 2013; Donovan et al. 2014, 2020) or marine shell or skeletal material (Kelley et al. 2003; Klompmaker et al. 2019), compared to those taking place on or within sediments. With increasing time depth, information about tracemaker identity and biology may be lost – for instance, mole-earthworm burrow associations would appear simply as those between large-diameter and small-diameter burrows without knowledge of the potential interactions involved, and an inability to distinguish between root traces and burrows by insects dependent on the roots themselves may make distinguishing between co-occurring composite or compound trace fossils difficult.

Temporal resolutions observed in horizons such as in the study paleosols and sediment horizons (decades to centuries or millennia) bridge the gap between those shorter timescales used in typical ecological studies, and those of deep time (tens of thousands to millions of years) more typical of paleontological data. There is an advantage in that the ecosystems and species in them are still very modern and well-understood but at the same time some traces may have already entered subfossil states where they are buried and beyond the reach of further disturbance, so that it may be possible to track what information would be lost or retained from them, deep into the geological record. Future studies in environments like these might be valuable in ground-truthing interpretations about biotic interactions so that they can inform paleoecological or ichnological reconstructions of ancient communities.
